# Lectures on Materia Medica and Therapeutics

**Published:** 1851-12

**Authors:** 


*Lectures on Materia Medica and Therapeutics.* By John Beck, M. D., late Professor in the College of Physicians and Surgeons, of New York. Prepared for the press by his friend, C. R. Gilman, M. D., Professor in the same College. New York: S. S. & W. Wood. 1851. 8vo. pp. 581.

This work will be heartily welcomed by the profession. The late lamented author was an able and most judicious teacher. His essays on the use of blisters and mercurials, in the diseases of children, evince the soundness of his instructions sufficiently for those who have not enjoyed the advantage of listening to his lectures at the College of Physicians and Surgeons in New York. The editor, Dr. Gilman, by preparing these lectures for the press, has not only discharged a pleasing duty to the memory of a deceased friend, but has rendered a valuable service to the profession.

A portion of the work is from the pen of the editor. He has supplied articles on *Anæsthetic Agents, Cod Liver Oil,* etc., subjects which were not embraced in the course of instruction by Dr. Beck. These additions render the volume more complete, and useful. The mechanical execution of the work is creditable to the enterprising medical publishers — the Woods.

*Hints to the People on the Profession of Medicine.* By Wm. Maxwell Wood, M. D., Surgeon U. S. Navy.

A stereotyped edition of the admirable essay by Dr. Wood, contained in the Sept. and Oct. numbers of this Journal, has been issued by Geo. H. Derby & Co., of this city. We would respectfully suggest to our readers that they may contribute to the interests of science and humanity, as well as promote their *private* interests, by purchasing copies of this essay for gratuitous circulation among their friends and neighbors.

We shall take occasion, at a future time, to pen some remarks on the *duty* and the *policy* of communicating for the public information respecting medical science and the medical profession, by means of judicious, well written tracts, like the essay by Dr. Wood. It will, at least, save not a little time, and colloquial trouble, to be able to respond to the various interrogatories addressed to physicians, by tendering a printed paper which the inquirer may read at his leisure.

The *getting up* of Dr. Wood's work is highly creditable to Buffalo typography. It is furnished at a low price, and will, we trust, have a very extensive circulation.

